# Pelvic vasectomy and its protective effects on rat testis function

**DOI:** 10.1186/s12610-025-00255-4

**Published:** 2025-03-07

**Authors:** Heng Yang, Yujun Chen, Xiaofeng Cheng, Jingxin Wu, Ruohui Huang, Biao Qian, Gongxian Wang

**Affiliations:** 1 Jiangxi Provincial Key Laboratory of Urinary System Diseases, Department of Urology, The First Affiliated Hospital, Jiangxi Medical College, Nanchang University, Nanchang, Jiangxi 330006 China; 2 Institute of Urology, First Affiliated Hospital of Gannan Medical University, Ganzhou, 341000 China; 3 Wuhan Integrated Traditional Chinese and Western Medicine Hospital (Wuhan First Hospital, Institute of Urology, Wuhan, 430000 China; 4 Institute of Urology, Donghu District, The First Affiliated Hospital of Nanchang University, No. 17 Yongwai Zhengjie, Nanchang, Jiangxi Province 330006 China

**Keywords:** Traditional Vasectomy, Modified Vasectomy, Epididymal Histopathology, Hormone Levels, Immunohistochemistry, Rats, Vasectomie traditionnellex, Vasectomie modifiée, Histopathologie épididymaire, Taux d’hormones, Immunohistochimie, Rats

## Abstract

**Background:**

Vasectomy is a commonly used male contraceptive method, but the choice of surgical technique can influence long-term reproductive health outcomes. Previous studies suggest that different vasectomy techniques may lead to varying degrees of tissue damage, oxidative stress, and endocrine dysfunction. However, there is limited research on how these techniques affect overall reproductive system function. Therefore, this study aims to evaluate and compare the effects of two vasectomy techniques on reproductive system parameters in rats.

**Methods:**

Twenty-four specific pathogen-free male Sprague–Dawley rats weighing 250–300 g were randomly divided into four groups: sham operation group, negative control group, traditional vasectomy group, and modified vasectomy group, with six rats in each group. Each group underwent specific vasectomy procedures, followed by a three-month recovery period. Experimental methods included hematoxylin and eosin staining, immunohistochemistry in the epididymis of rats, terminal deoxynucleotidyl transferase-mediated dUTP nick-end labeling for apoptosis detection, enzyme-linked immunosorbent assay for measuring serum hormone and oxidative stress markers, as well as tests for sexual behavior and anxiety-like behavior.

**Results:**

The modified vasectomy group exhibited improved epididymis morphology compared to the traditional vasectomy group. Immunohistochemistry demonstrated reduced levels of apoptosis in the modified vasectomy group, which was further corroborated by terminal deoxynucleotidyl transferase-mediated dUTP nick-end labeling staining, indicating lower cell death. Hormone analysis revealed stable levels in the modified vasectomy group, and oxidative stress markers indicated reduced stress responses. Behavioral test assessing sexual activity and anxiety level was consistent with these findings.

**Conclusion:**

Modified vasectomy techniques provide superior protection of reproductive system functionality in rats compared to traditional methods. These techniques reduce tissue damage, cell apoptosis, and oxidative stress while maintaining endocrine function, offering promising implications for clinical applications.

## Résumé

**Contexte:**

La vasectomie est une méthode contraceptive masculine couramment utilisée, mais le choix de la technique chirurgicale peut influencer les résultats à long terme en matière de santé reproductive. Des études antérieures suggèrent que différentes techniques de vasectomie peuvent entraîner des lésions tissulaires à des degrés divers, un stress oxydatif et un dysfonctionnement endocrinien. Cependant, il existe peu de recherches sur la façon dont ces techniques affectent le fonctionnement global du système reproducteur. Par conséquent, cette étude vise à évaluer et à comparer les effets de deux techniques de vasectomie sur les paramètres du système reproducteur chez le rat. Vingt-quatre rats Sprague-Dawley mâles ont été répartis au hasard en quatre groupes : le groupe d’opération simulée, le groupe témoin négatif, le groupe de vasectomie traditionnelle et le groupe de vasectomie modifiée, avec six rats dans chaque groupe. Chaque groupe a subi des procédures de vasectomie spécifiques, suivies d’une période de récupération de trois mois. Evaluations histologiques, stress oxydant, hormones sériques, et tests concernant la conduite sexuelle et le degré d’anxiété comportementale.

**Résultats:**

Le groupe de vasectomie modifiée a montré une morphologie de l’épididyme améliorée par rapport au groupe de vasectomie traditionnelle. L’immunohistochimie a montré des niveaux réduits d’apoptose dans le groupe de vasectomie modifiée, corroborés par une coloration terminale de marquage dUTP médiée par la désoxynucléotidyltransférase, indiquant une mort cellulaire plus faible. Les analyses hormonales ont révélé des niveaux stables dans le groupe de vasectomie modifiée, et les marqueurs de stress oxydatif ont indiqué une réduction des réponses au stress. Les tests comportementaux évaluant l’activité sexuelle et le niveau d’anxiété étaient cohérents avec ces résultats.

**Conclusions:**

Les techniques de vasectomie modifiée offrent une protection supérieure de la fonctionnalité du système reproducteur chez les rats par rapport aux méthodes traditionnelles. Ces techniques réduisent les lésions tissulaires, l’apoptose cellulaire et le stress oxydatif tout en maintenant la fonction endocrinienne, offrant des implications prometteuses pour les applications cliniques.

## Introduction

Vasectomy is a simple, minimally invasive, safe, and highly successful permanent sterilization method with virtually no complications [1]. It achieves sterility by ligating the vas deferens to block the expulsion of sperm from the body. Globally, over 100 million such procedures have been conducted, with at least 23 million vasectomies performed in China since the 1970s. According to data from Ostroski et al., 527,476 vasectomies were performed in the United States in 2015 alone [2]. However, there is still controversy regarding the impact of vasectomy on sperm production within the testis [3, 4], with a study suggesting severe damage to sperm [5] and others considering the effects to be negligible or non-existent [6–8]. This variation may be related to the method of ligation and the distance of the ligation site from the tail of the epididymis [9].

Research indicates that vasectomy may primarily affect sperm production within the testis through two mechanisms: Firstly, the surgery disrupts the blood-testis barrier, allowing antigens such as sperm to enter the bloodstream, triggering an autoimmune response that produces anti-sperm antibodies or enhances the activity of internal antibodies, thereby affecting sperm production within the testis. It is a type of immune-mediated systemic response [10–12]. Secondly, post-ligation, the testis continues to produce sperm and secrete testicular fluid, leading to changes in pressure near the testis end of the reproductive tract and within the testis itself, which are localized pressure-mediated changes [3].

In light of the potential adverse effects associated with traditional vasectomy procedures, our research team has developed a modified surgical method. This method optimizes the surgical steps and employs more refined techniques aimed at minimizing physical damage to the reproductive organs. Specific improvements include using minimally invasive techniques for precise cutting of the vas deferens and applying biocompatible materials to seal the vas deferens, reducing tissue reactions and inflammation. This modified approach is expected to effectively decrease damage to the seminiferous tubules and oxidative stress response, thereby protecting the testis’ and epididymis’ morphology and function and reducing cellular apoptosis.

The primary objective of this study is to systematically evaluate the impact of the modified vasectomy procedure on the reproductive system morphology, hormone levels, oxidative stress response, and sexual behavior in rats, compared to the traditional surgical method. Through detailed histological analysis, biochemical testing, behavioral assessments, and electron microscopy, this study aims to comprehensively understand the biological effects and potential physiological protective effects of the modified procedure. Scientifically, this research will fill a gap in the current literature regarding the efficacy of modified vasectomy methods and provide experimental evidence to support the development of less invasive contraceptive techniques. Clinically, these findings are expected to help physicians offer safer and more effective vasectomy options, reducing postoperative complications and improving the quality of life for male patients. Furthermore, this modified vasectomy technique may offer more options for patients considering vasectomy reversal, as it reduces the risk of reproductive organ damage, potentially increasing the success rate of reversal procedures.

## Materials and methods

### Experimental animals

Hunan Slaughter Laboratory Animal Co., Ltd provided 24 male SD rats (Rattus norvegicus) weighing 250–300 g and of specific pathogen-free (SPF) grade. The animals were maintained under a 12-h light/12-h dark cycle at a room temperature of 20–25℃ and a relative humidity of 50–65%. The housing environment was kept quiet, and bedding was changed 2–3 times weekly.

This study strictly adhered to national and institutional guidelines on using experimental animals. All animal experiments were approved by the respective Institutional Animal Care and Use Committee (Approval no.: 20045), ensuring the welfare of the experimental animals and minimizing their pain and discomfort. Before the start of the experiment, the rats underwent a one-week acclimation period to adapt to the housing conditions. All surgeries were performed under strict aseptic conditions using appropriate anesthesia methods to alleviate animal suffering. At the end of the experiment, all animals were humanely euthanized. Additionally, we ensured the scientific validity and rationale of the experimental design to make efficient use of each animal and avoid unnecessary repetition. We strive to ensure experimental animals’ ethical treatment and welfare through these measures while obtaining scientifically valid research data.

### Key reagents

The study employed the following high-quality reagents: TUNEL assay kit (C1088, Bioss) for detecting apoptosis in tissue sections. Bcl-2 Associated X Protein (Bax) antibody (50,599–2-lg, Proteintech) and B-cell lymphoma 2 (Bcl-2) antibody (26,593–1-AP, Proteintech) for detecting apoptosis-related proteins. Testosterone (T) synthase 3β-HSD (HSD3B1, A19266, ABclonal) and human epididymal protein 4 (HE4, bs-4626R, Bioss) for hormone analysis. Horseradish peroxidase-conjugated goat anti-rabbit IgG(H + L) (ZB-2301, Nakasugi Jinqiao) for secondary antibody labeling. DAB staining kit (CW0125, CWBIO) for immunohistochemical staining and neutral resin (CW0136, CWBIO) for tissue section sealing. Hematoxylin staining solution (AR1180-1, Boson Biotech) and eosin staining solution (G1100, Solarbio) for tissue staining. Malondialdehyde (MDA) and superoxide dismutase (SOD) assay kits (Nanjing Jiancheng Bioengineering Institute, China) for oxidative stress marker detection. BCA protein assay kit (Thermo Fisher Scientific, USA) for protein concentration normalization. Hormone ELISA kits: Testosterone (T) (Cayman Chemical, USA), Estradiol-17β(E2), Prostaglandin E2 (PGE2), prolactin (PRL), Luteinizing Hormone (LH), and Follicle-Stimulating Hormone (FSH) (Abcam, UK). The levels of anti-sperm antibodies (ASA) in serum were measured using a specific ELISA kit. Using these reagents ensured the accuracy of all assays throughout the experimental process.

### Study design

This study included 24 male Sprague–Dawley rats aged 8–10 weeks, weighing approximately 250–300 g. The rats were randomly divided into four groups: sham operation group, negative control group, traditional vasectomy group, and modified vasectomy group, with six rats in each group. Each group underwent specific vasectomy procedures. After a three-month recovery period, hematoxylin and eosin (H&E) staining, immunohistochemistry, terminal deoxynucleotidyl transferase dUTP nick-end labeling (TUNEL) assays, and enzyme-linked immunosorbent assay (ELISA) analysis were performed to evaluate serum hormone levels and oxidative stress markers. Behavioral tests were conducted to assess sexual activity and anxiety levels. Finally, transmission electron microscopy was used to examine the ultrastructure of testicular and epididymal tissues.

### Study location

All experiments were conducted at the First Affiliated Hospital of Nanchang University under strict laboratory and ethical guidelines.

### Study duration

The complete study cycle lasted 14 weeks.

### Inclusion and exclusion criteria

The inclusion criteria for preoperative rats were 8–10 weeks of age, a body weight of approximately 250–300 g, and good health with no apparent physiological or mental disorders. The postoperative inclusion criteria required that the rats had recovered well from surgery with no signs of infection or related complications. Pre- and postoperative exclusion criteria included mortality or significant adverse symptoms.

### Sample size calculation

The sample size was calculated using G*Power software. Assuming an effect size of 0.5, a significance level of 0.05, and a power of 0.8, the minimum required number of animals per group was six. It ensured sufficient statistical reliability for the study conclusions.

### Sampling methods

This study employed a completely randomized grouping method to allocate experimental animals. Specifically, all eligible animals were numbered, and a random number table or randomization software (such as the RAND function in Excel or the random grouping function in SPSS) was used to randomly assign 24 Sprague–Dawley rats into four experimental groups, with six rats in each group. The grouping results were finalized before the implementation of the experiments to avoid allocation bias.

### Study procedures

This study calculated the sample size using G*Power software, assuming an effect size of 0.5, a significance level of 0.05, and a statistical power of 0.8, determining that at least six rats per group were required. A total of 24 male Sprague–Dawley (SD) rats (*Rattus norvegicus*, provided by Viton Lihua), aged 8–10 weeks, were randomly divided into four groups: sham operation group, negative control group, traditional surgery group, and modified surgery group. Before surgery, the rats were fasted for 12 h, the abdominal hair was shaved, and the area was disinfected. Under isoflurane anesthesia, the sham operation group involved exposure of the vas deferens without ligation; the traditional surgery group used double ligation of the vas deferens; and the modified surgery group involved ligating the proximal end of the vas deferens while leaving the distal end open to reduce local pressure. Post-surgery, the rats were closely monitored for recovery, with analgesics and antibiotics administered as necessary. Three months after surgery, blood and tissue samples, including testes, epididymis, and vas deferens, were collected for (H&E staining, immunohistochemistry, TUNEL assays, and ELISA analysis. Additionally, anxiety behavior was assessed using the Elevated Plus Maze (EPM), and exploratory and mounting behaviors during sexual activity tests were recorded. All data were analyzed using SPSS (version 26.0) and GraphPad Prism (version 9.3.1).

### Study results and expected outcomes

This study primarily investigated the effects of different vasectomy techniques on the reproductive systems of male SD rats, including aspects such as morphology, hormone levels, oxidative stress responses, sexual behavior, and anxiety-like behavior. The study aims to demonstrate that the modified vasectomy technique offers superior surgical outcomes and physiological protective effects, providing scientific evidence for the clinical selection of safer and more effective vasectomy techniques.

### Vasectomy procedures

This study used 24 male SD rats (8–10 weeks old, 250-300g), randomly divided into four groups: sham surgery (SO), negative control (NC), traditional vasectomy (TV), and modified vasectomy (MV), with 6 rats per group. All rats were acclimatized for one week before the experiment. Before surgery, the rats were fasted for 12 h, and the surgical area was shaved and disinfected with 70% ethanol or iodine solution. Anesthesia was induced using 5% isoflurane, and once the rats were unresponsive to external stimuli, they were transferred to the surgical platform, where anesthesia was maintained at 1.5–2.0% isoflurane with 1L/min oxygen flow to ensure normal oxygen saturation.

A 1.5–2 cm incision was made in the lower abdomen to expose the testes and vas deferens. Depending on the group, the following procedures were performed: in the sham surgery group, the vas deferens were exposed without ligation; in the negative control group, no surgical procedure was performed; in the traditional vasectomy group (TV), the vas deferens was transected, and both ends were ligated; in the modified vasectomy group (MV), the vas deferens was transected with the proximal end (near the epididymis) ligated and the distal end (toward the urethra) left open. After surgery, the incision was closed with absorbable sutures or surgical glue, and the rats were placed in individual cages with food and water while their recovery and postoperative behavior were monitored [13, 14].

Postoperatively, the rats were closely monitored for recovery, and analgesics (e.g., ibuprofen) and antibiotics (e.g., vancomycin powder [HY-B0671, MedChemExpress] and tobramycin powder [HY-B0441, MedChemExpress]) were administered as needed to prevent infection [15]. After 3 months, all rats were anesthetized again, and the vas deferens were exposed to verify the ligation by injecting 2.0–3.0 mL of methylene blue solution into the abdominal end of the vas deferens. If the urine turned blue, the ligation failed; if no color change was observed, the ligation was successful.

### Collection of experimental animal specimens

After confirming the success of vasectomy using methylene blue staining, all experimental rats were euthanized according to standard procedures. The specific method involved intraperitoneal injection of 1% sodium pentobarbital (at a dose of 6 ml/kg) for anesthesia. Once the anesthesia took effect, euthanasia was performed under sterile conditions. Subsequently, through the previously made surgical incision, the bilateral testis, epididymis, and vas deferens tissues were removed from each rat [16]. These tissue specimens were used for subsequent pathological and biochemical analyses.

### Epididymal histology

After successfully establishing the model, epididymis tissue was collected from anesthetized rats and fixed with 4% paraformaldehyde for 24 h. The tissue was then dehydrated using a graded ethanol series, followed by clearing with xylene for 20 min and embedding in paraffin. Before sectioning, the blade edge was checked under a magnifying glass for smoothness, and the block was properly aligned on the specimen stage. The blade angle was set to approximately 15°, and sectioning was done at 5 µm thickness. The paraffin sections were heated, dewaxed, and hydrated. The sections were stained with hematoxylin for 3 min, differentiated in hydrochloric acid–ethanol solution for 15 s, rinsed with water, blued for 15 s, and stained with eosin for 3 min. Finally, the sections were dehydrated, cleared, and mounted [17]. After staining, the epididymal tissue was observed under a microscope to assess the effects of surgery on the tissue structure.

### Immunohistochemistry

The expression of Bax (1:100, MA5-35,342, ThermoFisher Scientific), Bcl-2 (1:200, PA5-27,094, ThermoFisher Scientific), HE4 (1 µg/ml, PA5-80,227, ThermoFisher Scientific), and 3βHSD (1:200, PA5-106,895, ThermoFisher Scientific) proteins in epididymal tissue was detected using immunohistochemistry [18]. The detailed steps are as follows:

First, paraffin sections were heated to melt paraffin wax and adherence of sections to glass slide, then deparaffinization and antigen hydrolysis in a water bath. Antigen retrieval was performed using the heat-induced epitope retrieval (HIER) method, where sections were heated in sodium citrate buffer (pH 6.0) using a microwave at high power until boiling to restore antigenicity. The sections were then incubated in a blocking solution to minimize nonspecific binding.

Subsequently, primary and secondary antibodies (1:200, 31,460, ThermoFisher Scientific) were sequentially applied. After appropriate incubation and washing, the sections were subjected to staining using DAB solution (P0203, Beyotime) for 3 min for color development, followed by counterstaining with hematoxylin (ST2067, Beyotime) for 4 min. Finally, the sections were dehydrated, cleared, and mounted. The expression of Bax, Bcl-2, HE4, and 3βHSD in epididymal tissue was observed under a microscope to assess the impact of surgery on apoptosis-related proteins.

### TUNEL staining

To evaluate the effects of different surgical treatments on apoptosis in rat epididymis cells, researchers used TUNEL staining [19]. In the experiment, paraffin sections of the testes and epididymis tissues were prepared from rats in each group. The paraffin sections were dewaxed in xylene, hydrated through a series of ethanol gradients, and subjected to antigen retrieval in a citrate buffer. After retrieval, the sections were rinsed with PBS and stained using a TUNEL detection kit (C1091, Beyotime)according to the manufacturer’s instructions, incubating for 1 h, followed by PBS washes to remove unbound probes. The cell nuclei were counterstained with DAPI, incubated for 10 min, and rinsed again with PBS. The stained sections were observed under a fluorescence microscope (BX43, OLYMPUS), and images of TUNEL-positive cells (green fluorescence) and DAPI-stained nuclei (blue) were captured. Five random fields were selected from each group to count the number of TUNEL-positive cells. The experiment was repeated three times to ensure data reliability.

### ELISA/Biochemical assay

After successfully establishing the model, blood samples from rats were collected via the abdominal aorta, and serum was separated through centrifugation. To quantify the levels of interleukin-1 (IL-1) (ab255730, Abcam, UK), interleukin-2 (IL-2), (ab221834, Abcam, UK), interleukin-6 (IL-6)(ab234570, Abcam, UK) testosterone (ab285350, Abcam, UK) and ASA(ab242796, Abcam, UK) in serum, ELISA was performed [20, 21]. First, standard wells, blank wells, and sample wells were prepared. Each standard well was loaded with 50 μL of standards at varying concentrations, blank wells received no sample or enzyme reagents, and sample wells were loaded with 50 μL of test samples. Subsequently, 100 μL of enzyme reagent was added to each well (excluding blank wells), and the plate was sealed with a sealing membrane and incubated at 37 ℃ for 60 min. After incubation, the plate was washed to remove unbound antibodies, followed by a color development reaction, which was then terminated. Finally, each well’s absorbance (A value) was measured at 450 nm using a microplate reader, with blank wells as the reference. The results for each sample group were quantitatively analyzed.

The levels of E2 (ab285291, Abcam, UK), LH (E-EL-R0026, Elabscience), PGE2 ((ab316263, Abcam, UK),PRL (ab214572, Abcam, UK) and FSH (E-EL-R0391, Elabscience) were measured using specific ELISA kits. The procedures were similar to those for testosterone detection, with each test including sample, standard, and blank control groups. The concentrations of each hormone were calculated using the corresponding standard curve. Results for E2 and PGE2 were expressed in pmol/L, while IL-1, IL-2, IL-6, T, PRL, and ASA were expressed in pg/mL, and LH and FSH were expressed in mIU/L. The intra-assay coefficient of variation (CV) for all measured parameters was below 10%, and the inter-assay CV was below 15%.

### Oxidative stress indicators

To evaluate the effects of different surgical methods on oxidative stress in rat testicular and epididymal tissues, we measured the levels of malondialdehyde (MDA) and superoxide dismutase (SOD) in this study. Testicular and epididymal tissues were immediately collected from each group of rats (6 rats per group, 24 rats in total) upon euthanasia and rapidly frozen at −80 ℃. For biochemical analysis, approximately 100 mg of tissue was homogenized in cold PBS (pH 7.4) at a ratio of 1:9. The homogenate was then centrifuged at 12,000 g for 15 min at 4℃, and the supernatant was collected for subsequent analysis.

For MDA measurement, 100 µL of sample was mixed with 100 µL of working solution, heated in a water bath at 95°C for 40 min, and then cooled. The absorbance (A value) was measured at 532 nm. MDA concentrations were calculated using a standard curve and expressed as nanomoles per milligram of protein (nmol/mg protein). For SOD activity, 100 µL of sample was mixed with the working solution, incubated at 37°C for 30 min, and the absorbance was measured at 450 nm. SOD activity was calculated based on a standard curve and expressed as units per milligram of protein (U/mg protein). MDA and SOD levels were normalized to protein concentrations [22, 23].

### Sexual behavior test

In order to assess the impact of surgery on rat sexual behavior, we conducted a spontaneous sexual behavior test. In the four groups, one male rat was placed with two estrous female rats in a standard experimental cage, and each group was observed for 30 min under the same conditions. The experimental environment was kept quiet, with uniform lighting, and efforts were made to minimize external disturbances. The test was performed during the light cycle’s middle, corresponding to the rats’ natural active period. Sexual motivation and activity were evaluated by observing and recording the following behavioral indicators: exploratory behaviors (e.g., sniffing, climbing) and mounting posture [24]. Two independent experimenters manually recorded and analyzed behavioral data using behavioral analysis software (Noldus EthoVision XT, Noldus Information Technology, Netherlands).

### Anxiety behavior test

The anxiety behavior test was conducted using the EPM, consisting of two open and two closed arms, with a central platform elevated 50 cm above the ground. Each rat was placed on the central platform facing one of the open arms, and the test lasted for 5 min. The environment remained quiet during the test, and any disturbances were avoided. Rat behavior was tracked using a video tracking system (ANY-maze, Stoelting Co., USA), including the time spent in open and closed arms and the number of entries into the open arms. The longer time spent in the open arms and more entries into the open arms indicate lower anxiety levels [25].

### Statistical analysis

Statistical analysis was performed using SPSS 20.0 software. All experiments were conducted in triplicate, and quantitative results were expressed as mean ± standard deviation (X ± S). An independent samples t-test was used for comparisons between two groups, while a one-way analysis of variance (ANOVA) was used for comparisons between multiple groups, followed by the Student–Newman–Keuls (S–N-K) method for pairwise comparisons. GraphPad Prism 6 was used to create statistical charts with a significance level of 0.05. The statistical methods of the study were reviewed by a biostatistician at Nanchang University.

## Results

### Gross morphology

**Fig. 1:**
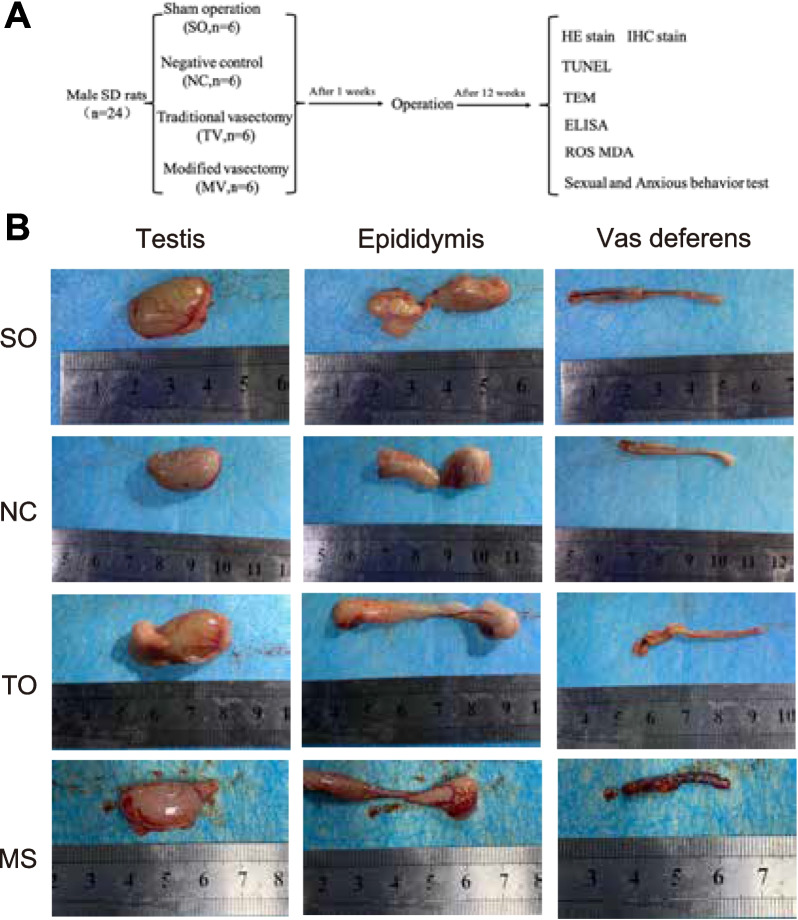
Rat Vasectomy Modeling and Post-Operative General Status and Morphological Observation. Note: (**A**) Schematic illustration of the vasectomy procedure in rats, outlining the experimental groups, timeline, and detection methods used. **B** Representative images of gross morphology of reproductive organs in rats from each group. The image shows the testis morphology of the negative control, sham operation, traditional, and modified vasectomy groups. The testes of the traditional vasectomy group are significantly enlarged and have severe adhesions with surrounding tissues, with serious changes in the morphology of the epididymis and the proximal vas deferens. The morphology of the testes and epididymis in the modified vasectomy group is essentially consistent with the negative control group, with no apparent adhesions. The images represent the typical morphological characteristics of rats from each group. SO: sham operation group, NC: negative control group, TV: traditional vasectomy group and MV: modified vasectomy group

This study successfully established four different vasectomy models (Fig. 1A). Following the surgery, all rats were maintained for three months to observe the long-term effects on the morphology of their reproductive systems. Through gross observation and anatomical examination, it was found that the testes of rats in the negative control group, sham operation group, and modified vasectomy group appeared normal, with no significant abnormalities or adhesions with surrounding tissues. It indicates that the testes’ integrity and surrounding tissues in these three groups remained unchanged and were not significantly affected. However, in the traditional vasectomy group, there was a noticeable increase in the size of the testes, and during dissection, severe adhesions between the testes and epididymis and the surrounding tissues were observed. This condition suggests this surgical method may have caused significant reactive changes and inflammation in the local tissues (Fig. 1B). The results demonstrate that the modified vasectomy group was superior in maintaining the morphology of the testes compared to the traditional vasectomy group, which was more prone to pathological changes in the testes and epididymis.

### Histology of epididymis

**Fig. 2:**
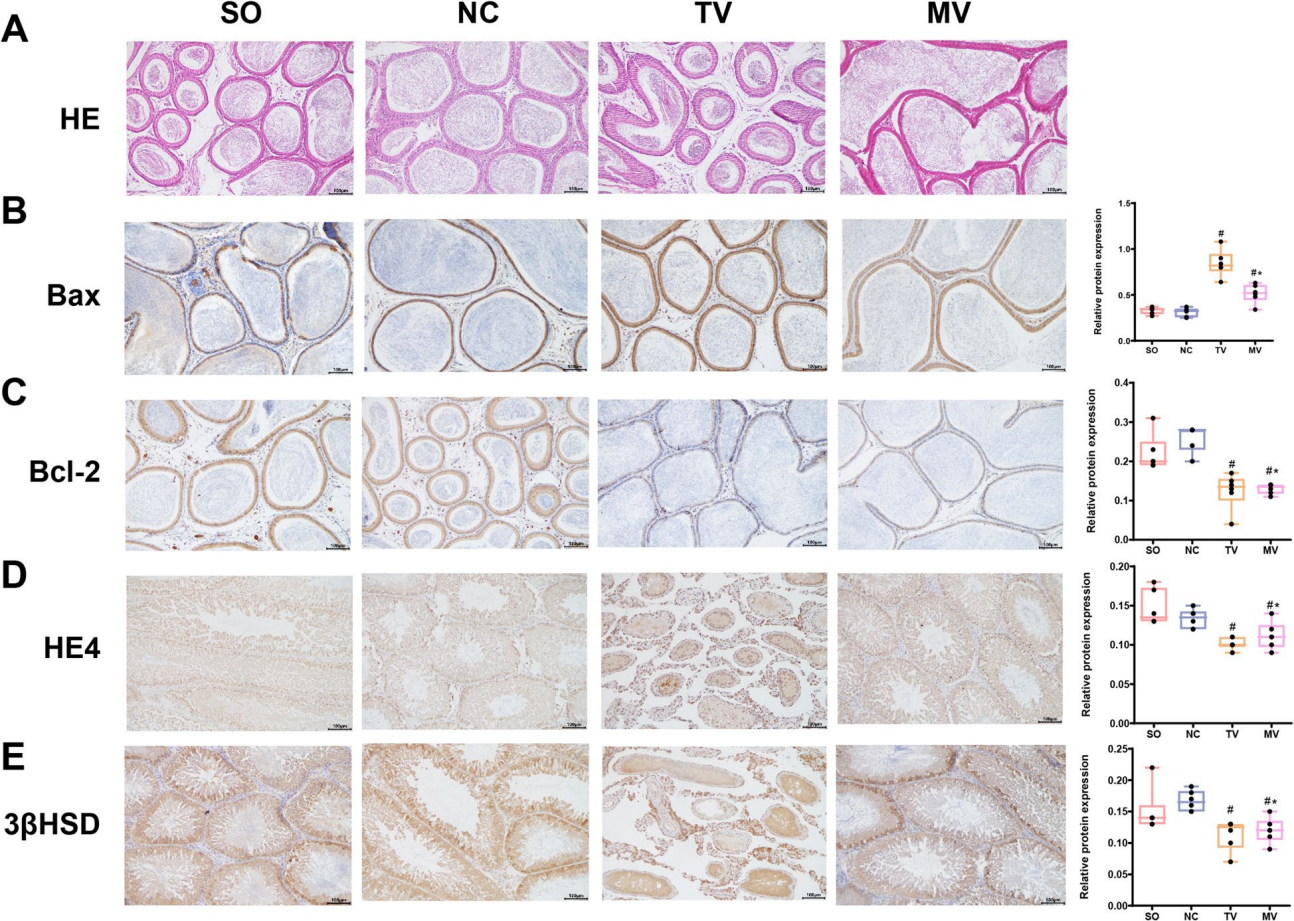
Histological and Immunohistochemistry Analysis of Rat Epididymal Tissue in Different Experimental Groups. Note: (**A**) Representative hematoxylin–eosin stained images of epididymal tissues from each group of rats. The traditional vasectomy and modified vasectomy groups exhibited significant swelling and sclerosis in the epididymal tissues, with the modified vasectomy group showing less severe tissue damage. In contrast, the sham operation group maintained a relatively normal epididymal structure, with only mild tissue reactions and no significant swelling or sclerosis. Scale bar = 100 μm. **B** Representative immunohistochemical images of Bax protein in epididymal tissues. Bax expression was significantly elevated in both the traditional vasectomy and modified vasectomy groups, with a more pronounced increase in the traditional vasectomy group, indicating heightened apoptotic activity in the epididymal tissues of this group. Scale bar = 100 μm. **C** Representative IHC images of Bcl-2 protein (anti-apoptotic) in epididymal tissues. Bcl-2 expression decreased to varying degrees in both the modified and traditional vasectomy groups, with the most substantial reduction observed in the traditional vasectomy group, further suggesting greater apoptotic stress in the epididymal tissues of this group. Scale bar = 100 μm. **D** Representative IHC images of HE4 protein in epididymal tissues. HE4 protein levels were reduced in both the traditional vasectomy and modified vasectomy groups, with a more significant decline in the traditional vasectomy group, indicating greater damage to the epididymis. Scale bar = 100 μm. **E** Representative IHC images of 3β-HSD protein in epididymal tissues. 3β-HSD protein levels were reduced in both the traditional vasectomy and modified vasectomy groups, with the traditional vasectomy group showing the most pronounced decline, indicating greater epididymal damage. Scale bar = 100 μm. (*P < 0.05 vs. sham operation; #*P* < 0.05 vs. traditional vasectomy). Each group included six rats. Statistical analyses were performed using SPSS 20.0 software, and quantitative data were presented as X ± S. Independent sample t-tests were used to compare the two groups. GraphPad Prism 6 was used to create statistical graphs. Significance level = 0.05. The groups are defined as follows: SO: sham operation group; NC: negative control group; TV: traditional vasectomy group; MV: modified vasectomy group

H&E staining (Fig. 2A) was used to observe and analyze the pathological changes in the epididymal tissue of each rat group. The results showed that the rats’ epididymal tissues exhibited noticeable swelling and hardening in both the traditional and modified vasectomy groups. Particularly during periods of inflammation, pathological dilation of the epididymis ducts occurred, which was especially pronounced in the traditional vasectomy group. In contrast, the epididymal tissue of rats in the sham operation group maintained a relatively normal structure, with only mild tissue reactions and no significant swelling or hardening observed. These findings suggest that traditional operation causes more severe damage to epididymal tissues, whereas the modified surgery, although also causing some pathology, overall resulted in less tissue damage.

### Immunohistochemistry

Immunohistochemistry analysis further revealed changes in the expression of Bax and Bcl-2 proteins in the epididymal tissues of rats from each group (Fig. 2B, C). Compared to the negative control group, the sham operation group showed no significant changes in Bax protein expression in the epididymal tissue, suggesting minimal impact on cell apoptosis. However, in both the modified and traditional operation groups, Bax expression was significantly elevated, with the traditional vasectomy group showing a more pronounced increase, indicating higher apoptotic activity in the epididymal tissue of this group. Conversely, the expression of Bcl-2 (an anti-apoptotic protein) decreased to varying degrees in both groups, with the traditional vasectomy group exhibiting the largest decrease, further suggesting that the epididymal tissue in this group was under greater apoptotic stress. Compared to the negative control, the sham operation group showed no significant changes in HE4 and 3β-HSD expression, indicating minimal epididymal damage. However, both the modified and traditional vasectomy groups exhibited damage to the epididymis, with the traditional vasectomy group showing the greatest reduction (Fig. 2D, E). These findings indicate that although the modified surgery provides some protective effects, the traditional operation significantly impacts apoptosis and epididymal tissue damage.

**Fig. 3:**
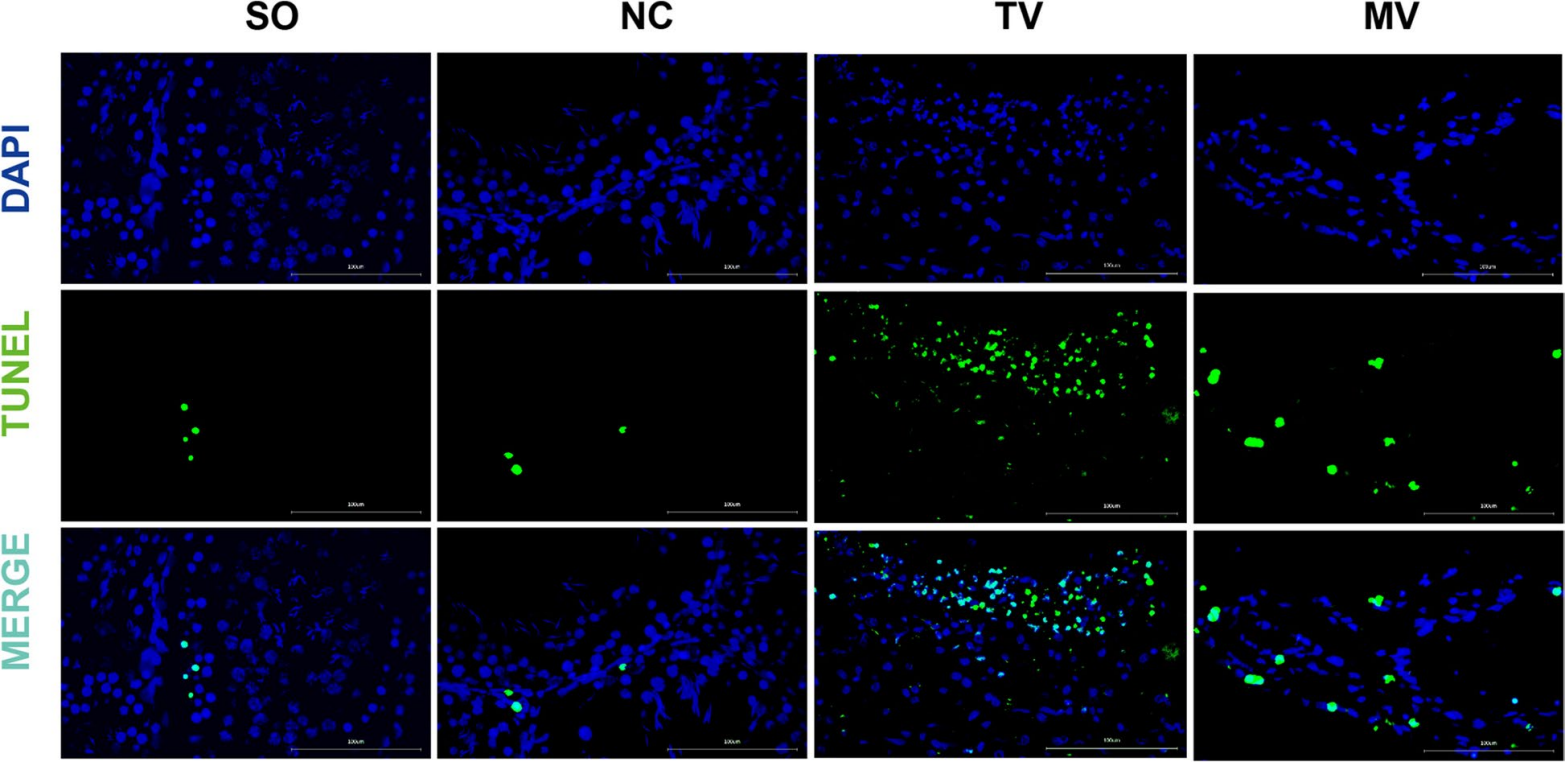
TUNEL Assay Reveals Cellular Apoptosis In Epididymal Tissue Across Different Experimental Groups. Note: Representative TUNEL staining images of epididymal tissues from each group. DAPI: DAPI staining highlights the blue nuclei morphology of epididymal tissues. While a few TUNEL-positive cells were observed in the modified vasectomy group, most nuclei remained intact, indicating a low apoptosis rate. In contrast, the traditional vasectomy group exhibited widespread distribution of TUNEL-positive cells, particularly concentrated in lesion areas. The nuclear morphology showed prominent apoptotic features, reflecting severe tissue damage and apoptosis induced by this surgical approach. TUNEL: TUNEL staining marks apoptotic cells in green. The proportion of TUNEL-positive cells in the modified vasectomy group was significantly lower than in the traditional vasectomy group. In the negative control and sham operation groups, very few TUNEL-positive cells were observed, and DAPI staining showed normal nuclear structures with minimal signs of apoptosis. Merge: Merged images of DAPI and TUNEL staining. Scale bar = 100 μm. The groups are defined as follows: SO: sham operation group; NC: Negative control group; TV: traditional vasectomy group; MV: modified vasectomy group

TUNEL staining results showed that the proportion of TUNEL-positive cells in the epididymal tissue of the modified vasectomy group was significantly lower than that in the traditional vasectomy group, suggesting that the modified procedure effectively reduced surgery-induced cell apoptosis. In both the negative control and sham operation groups, there were very few TUNEL-positive cells, and DAPI staining showed normal nuclear structure with almost no signs of apoptosis. Although many TUNEL-positive cells were present in the modified vasectomy group, the overall quantity was low, and most nuclei remained intact, indicating a lower apoptosis rate. In contrast, in the traditional vasectomy group, TUNEL-positive cells were widely distributed, particularly concentrated in the affected areas, and the nuclear morphology displayed clear apoptotic features, reflecting more severe tissue damage and cell apoptosis caused by this procedure (Fig. 3). These results suggest that the modified surgery has a clear advantage in reducing cell apoptosis and mitigating epididymal tissue damage, providing potential clinical value.

### Hormonal analyses

**Fig. 4:**
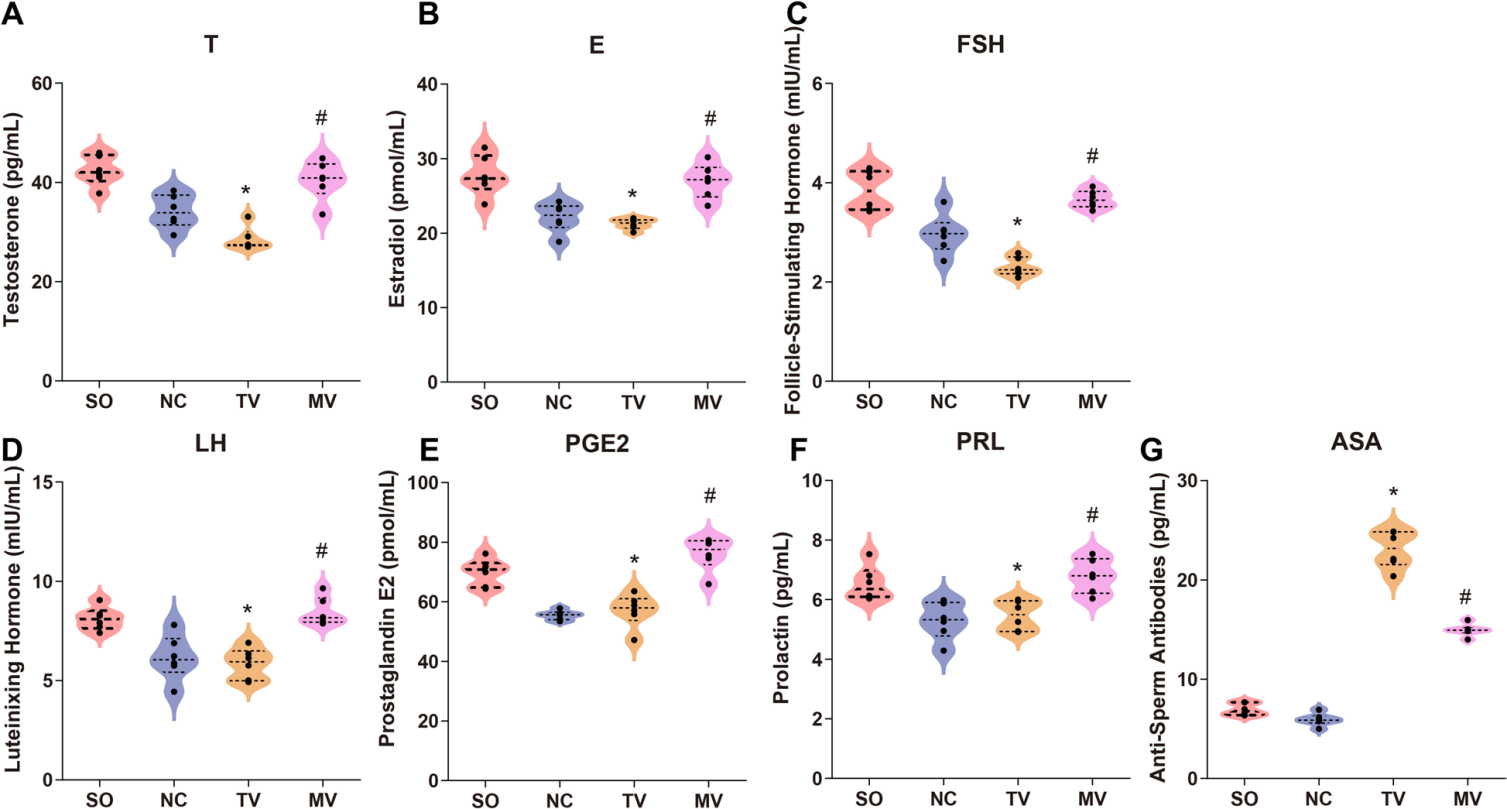
Serum Hormone Levels in Rats Following Different Vasectomy Procedures. Note: (**A**) Serum testosterone (T) levels: Rats in the modified vasectomy group exhibited significantly higher serum testosterone levels compared to the traditional vasectomy group, indicating that the modified vasectomy procedure better preserves testicular function. **B** Serum estradiol (E) levels: Serum estradiol levels in the modified vasectomy group were closer to those in the negative control group, whereas levels in the traditional vasectomy group were significantly reduced, suggesting that the traditional vasectomy procedure may cause greater endocrine disruption. **C** Serum follicle-stimulating hormone (FSH) levels: The modified vasectomy group showed higher serum FSH levels, reflecting relatively stable endocrine function, while the traditional vasectomy group displayed a marked decrease. **D** Serum luteinizing hormone (LH) levels: Serum LH levels were higher in the modified vasectomy group, indicating relatively stable endocrine function, whereas levels in the traditional vasectomy group were significantly lower. **E** Serum prostaglandin E2 (PGE2) levels: PGE2 levels were significantly elevated in the modified vasectomy group, suggesting that this procedure may be more effective in controlling local inflammation. **F** Serum prolactin (PRL) levels: PRL levels in the modified vasectomy group were similar to those in the negative control group. **G** Serum anti-sperm antibody levels, with the modified surgery group showing significantly lower serum anti-sperm antibody levels than the traditional surgery group (*P < 0.05 vs. sham operation; #*P* < 0.05 vs. traditional vasectomy). Statistical analyses were performed using SPSS 20.0 software. Each group included six rats, and quantitative results are presented as X ± S. Independent sample t-tests were used for comparisons between groups. GraphPad Prism 6 was used to create statistical graphs. The significance level was set at 0.05. Group definitions: SO, sham operation group; NC, negative control group; TV, traditional vasectomy group; MV, modified vasectomy group

In analyzing serum hormone levels in rats, the modified surgery demonstrated better protection of endocrine balance. ELISA/biochemical analysis results indicated that serum T levels in the modified surgical group were significantly higher than in the traditional vasectomy group, suggesting that the modified surgery has certain advantages in maintaining testis function (Fig. 4A). In addition, estradiol (E) levels in the modified vasectomy group were similar to those in the negative control group, whereas E levels in the traditional vasectomy group were significantly reduced, suggesting that the traditional vasectomy procedure may cause greater disruption to the endocrine system (Fig. 4B). Meanwhile, serum FSH and LH levels were higher in the modified vasectomy group, indicating relatively stable endocrine function, whereas these levels were significantly reduced in the traditional vasectomy group (Fig. 4C-D). In the measurement of FSH, there was no significant difference between the modified and traditional vasectomy groups (Fig. 4C). In the measurement of PGE2, levels were significantly elevated in the modified vasectomy group, suggesting it may be more effective in controlling local inflammation (*P* < 0.05) (Fig. 4E). In addition, prolactin (PRL) levels in the modified vasectomy group were similar to those in the negative control group (Fig. 4F). Further sperm antibody detection results indicated that the ASA levels in the modified surgery group were significantly lower than those in the traditional surgery group (Fig. 4G). Overall, the modified surgery better maintained endocrine balance and reduced the negative impacts caused by surgery.

### Oxidative stress indicators

**Fig. 5:**
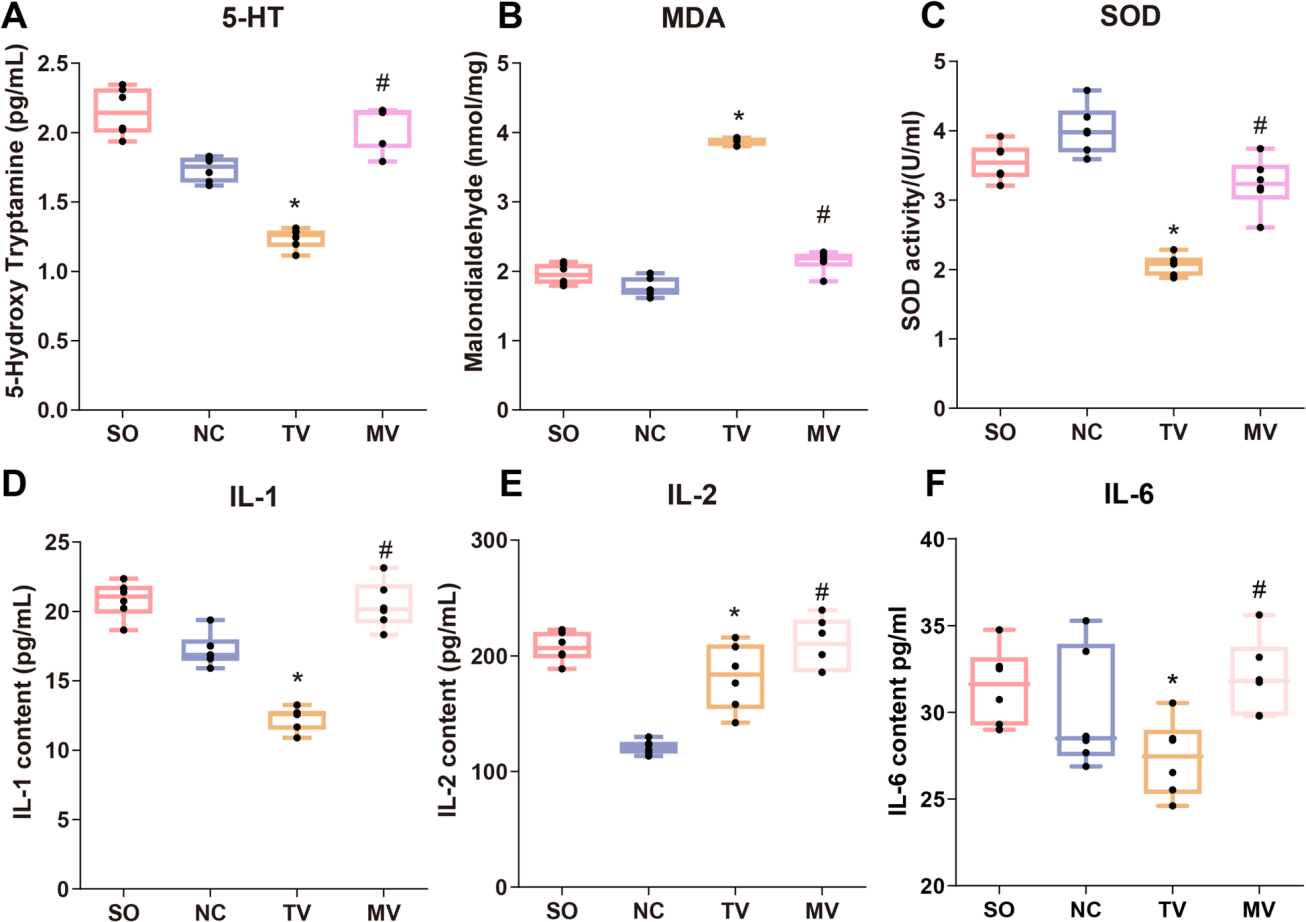
Oxidative Stress Markers and Inflammatory Factors in Rat Serum Following Different Vasectomy Surgeries. Note: (**A**) Serum serotonin (5-HT) levels: The 5-HT levels in the MV group were lower than those in the traditional vasectomy group. **B** Serum malondialdehyde (MDA) levels: The MDA levels in the testicular and epididymal tissues of the modified vasectomy group were lower than those in the traditional vasectomy group. **C** Serum superoxide dismutase (SOD) activity: The SOD activity in the testicular and epididymal tissues of the modified vasectomy group was higher than that in the traditional vasectomy group. (D-F) Serum interleukin-1 (IL-1), interleukin-2 (IL-2), and interleukin-6 (IL-6) levels: The serum levels of IL-1, IL-2, and IL-6 were lower in the modified vasectomy group compared to the traditional vasectomy group, reflecting more severe tissue damage and inflammatory responses in the traditional vasectomy group. (*P < 0.05 vs. sham operation; #*P* < 0.05 vs. traditional vasectomy). Statistical analyses were performed using SPSS 20.0 software. Each group included six rats, and quantitative results are presented as X ± S. Independent sample t-tests were used for comparisons between groups. GraphPad Prism 6 was used to create statistical graphs. The significance level was set at 0.05. Group definitions: SO, sham operation group; NC, negative control group; TV, traditional vasectomy group; MV, modified vasectomy group

ELISA/biochemical analysis results showed that serotonin (5-HT) levels in the modified vasectomy group were lower than in the traditional group (*P* < 0.05) (Fig. 5A). In the measurement of oxidative stress markers, the levels of MDA were lower, and the activity of SOD was higher in the epididymis tissues of rats in the modified vasectomy group, indicating a milder oxidative stress response in these rats. It suggests that the modified surgery effectively reduced the production of free radicals and oxidative tissue damage triggered by the surgery. In contrast, the traditional surgery group exhibited significantly elevated MDA levels and reduced SOD activity, reflecting a stronger oxidative stress response. This response could be due to local tissue damage and inflammation caused by double-ligation, further demonstrating the advantage of the modified surgery in reducing oxidative stress (Fig. 5B-C). Similarly, the levels of IL-1, IL-2, and IL-6 in the serum of rats from the modified vasectomy group were lower than those in the traditional surgery group, indicating that rats in the traditional group may have experienced more severe tissue damage and inflammatory responses (*P* < 0.05) (Fig. 5D-F).

### Sexual and anxiety behavior

**Fig. 6:**
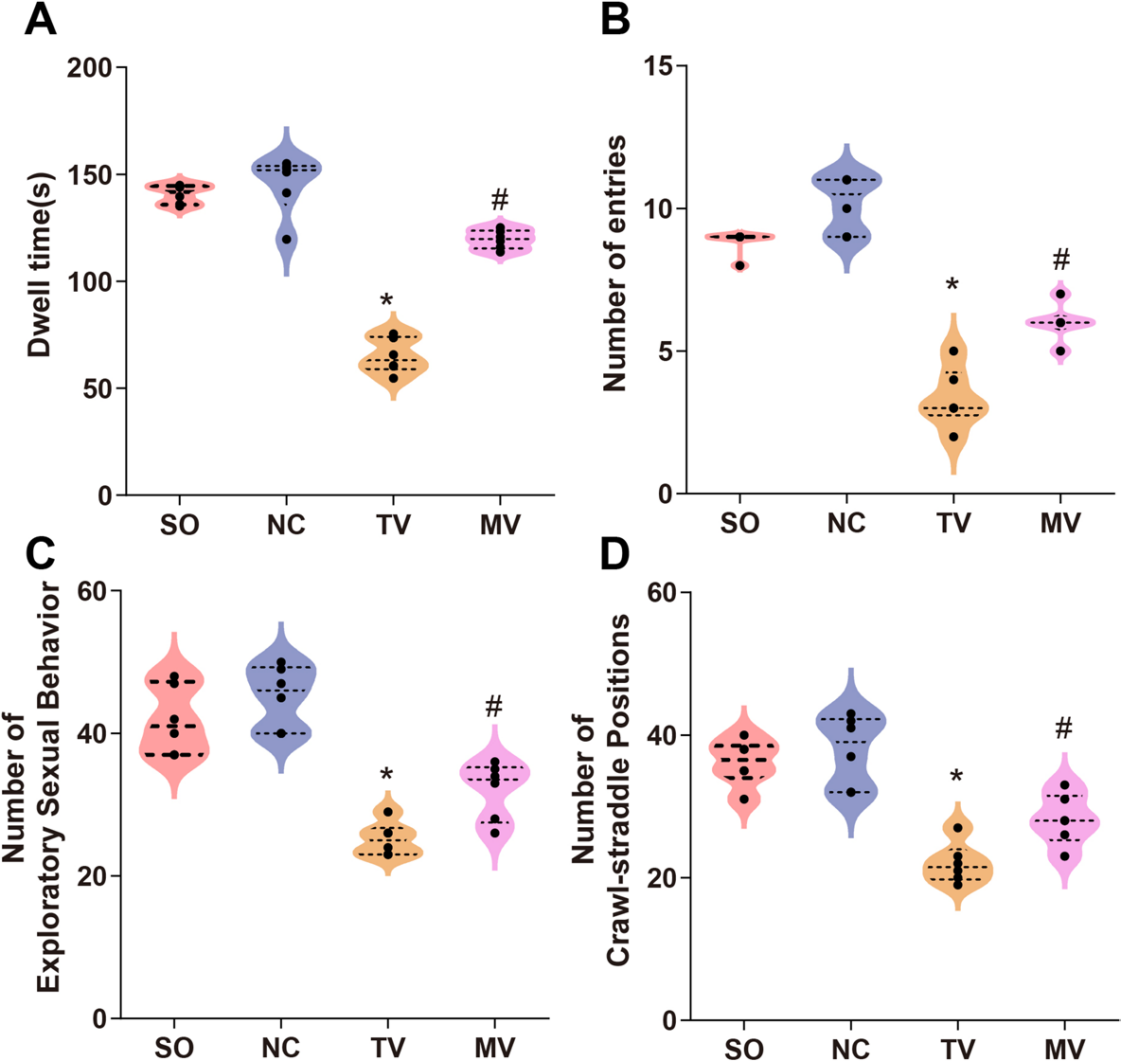
Behavioral Assessment in Different Experimental Rat Groups. Note: (**A**) Elevated plus-maze test: Open arm duration. Rats in the modified vasectomy group exhibited lower anxiety levels, as evidenced by significantly longer durations spent in the open arms, indicating reduced anxiety and psychological stress. In contrast, rats in the traditional vasectomy group displayed marked anxiety behaviors, spending significantly more time in the closed arms. **B** Elevated plus-maze test: Open arm entries. Rats in the modified vasectomy group showed lower anxiety levels, as demonstrated by an increased number of open-arm entries, indicating reduced anxiety and psychological stress. Conversely, rats in the traditional vasectomy group exhibited fewer entries into the open arms, reflecting heightened anxiety. **C**-**D** Sexual behavior test: Rats in the modified vasectomy group displayed significantly higher sexual activity, characterized by increased exploratory behavior (**C**) and frequent mounting postures (**D**). These indicators were similar to those observed in the negative control group and sham operation groups, suggesting that sexual desire and physiological functions were largely preserved in the modified vasectomy group. Furthermore, the modified vasectomy group outperformed the traditional vasectomy group in the sexual behavior test. (*P < 0.05 vs. sham operation; #*P* < 0.05 vs. traditional vasectomy). Statistical analyses were performed using SPSS 20.0 software. Each group included six rats, and quantitative results are presented as X ± S. Independent sample t-tests were used for comparisons between groups. GraphPad Prism 6 was used to create statistical graphs. The significance level was set at 0.05. Group definitions: SO, sham operation group; NC, negative control group; TV, traditional vasectomy group; MV, modified vasectomy group

In the EPM test for anxiety behaviors, rats from the modified vasectomy group exhibited lower anxiety levels. It was evidenced by longer durations spent in the open arms and more frequent entries into these arms, indicating lower anxiety and psychological stress in the rats. In contrast, the traditional vasectomy group displayed pronounced anxious behaviors, spending more time in the closed arms and less frequently entering the open arms (Fig. 6A, B). This result may be related to the greater physiological and psychological stress caused by traditional operations. By reducing surgical trauma and local tissue stress responses, the modified vasectomy group effectively lowered the rats’ anxiety levels, further demonstrating the psychological protective effects of the modified surgical approach.

In the sexual behavior test, rats in the modified vasectomy group exhibited significantly higher sexual activity, characterized by increased exploratory behavior and frequent mounting postures. These behavioral indicators were comparable to those observed in the negative control group and the sham operation group, suggesting that sexual drive and physiological functions in the modified vasectomy group were largely preserved. Furthermore, the modified vasectomy group outperformed the traditional vasectomy group in the sexual behavior test. The traditional vasectomy group, due to the physiological stress and tissue damage caused by bilateral vas deferens ligation, displayed markedly reduced sexual activity, which included a significant decrease in exploratory behavior and mounting posture frequency. The reduced sexual behavior in the traditional vasectomy group may be associated with increased local pressure and tissue lesions in the epididymis and testis caused by bilateral vas deferens ligation. In contrast, the modified surgical method, by maintaining an open state in the vas deferens between the epididymis and testis, alleviated local pressure and better preserved sexual drive and physiological functions (Fig. 6C-D).

## Discussion

Vasectomy is a common method of male contraception, and although highly safe and reliable, its long-term effects on the reproductive system remain a focus of clinical and research interest [26, 27]. Previous studies have mainly concentrated on changes in reproductive function, hormone levels, and the impact on sperm production following the surgery [28, 29]. However, there has been relatively little advancement in the surgery techniques and a deep analysis of their physiological impacts. This study introduces a modified vasectomy technique aimed at reducing the negative effects on the reproductive system, particularly in terms of cellular damage and hormonal imbalances, marking a significant departure from earlier studies that focused primarily on the consequences of the procedure.

The modified pelvic vasectomy technique, for the first time, exposes the testes and bilateral vas deferens, cutting the vas deferens at the terminal segment within the pelvis while keeping the epididymal end of the vas deferens open, only ligating near the urethral end. This method has not been widely reported both domestically and internationally. This new technique helps to alleviate the increased epididymal pressure caused by traditional vasectomy procedures, thus reducing the incidence of complications such as congestive epididymitis and painful sperm granuloma [30–32]. This study compared the effects of traditional vasectomy and modified vasectomy. The results indicate that the modified procedure offers significant advantages in reducing epididymal tissue damage, maintaining testicular and epididymal function, stabilizing hormone levels, and alleviating oxidative stress. Compared to the traditional method, the modified surgery significantly reduces the pressure on the epididymis caused by sperm, decreases epididymal lesions due to sperm accumulation, and improves the physiological and behavioral performance of the animals [29, 33, 34]. However, despite the promising results, the modified surgery’s practical feasibility and long-term effects should still be considered. Future research should further validate its applicability in larger sample sizes and under different conditions.

According to the study by Lutai et al., in conventional vasectomy procedures, no significant difference was observed in the expression of Bcl-2 in spermatogenic cells, while Bax expression significantly increased, leading to a notable increase in the number of apoptotic spermatogenic cells [35]. Our study obtained similar results: compared to the negative control group, the sham operation group showed no significant difference; both the modified vasectomy group and the traditional vasectomy group exhibited increased expression of Bax, especially pronounced in the traditional vasectomy group; expression of Bcl-2 decreased in both the modified and traditional vasectomy groups, with a greater decrease observed in the traditional vasectomy group.

Regarding hormone levels, the rats in the modified vasectomy group exhibited relatively stable T, E2, and other hormones, contrasting with the significant fluctuations in the traditional vasectomy group. This finding supports the hypothesis that reducing damage to reproductive organs can maintain hormonal stability, thereby supporting normal reproductive function. Previous research has primarily focused on the short-term changes in hormone levels following ligation [36], while our study provides a potential strategy to alleviate this issue through surgical improvement.

Behavioral tests showed that rats in the modified vasectomy group exhibited lower anxiety behaviors and higher sexual activity levels. It is consistent with some studies, which found that vasectomy might affect an animal’s behavioral performance [37]. This study further demonstrates that reducing physiological stress caused by surgery can lead to positive changes at the behavioral level, which is significant for understanding the impact of surgery on overall physiological and behavioral health.

### Limitations of the study

Despite this study providing beneficial prospective data for the clinical application of modified vasectomy, there are several limitations. Firstly, the study sample size is relatively small, with only 6 rats per group, which may affect the statistical significance of the results and their generalizability to a broader population. Secondly, the experimental model used rats, not humans, so the direct applicability of the study results to humans must be treated with caution. Furthermore, the study only lasted three months, and data on the long-term effects of the surgery still need to be provided. Therefore, future research should expand the sample size, extend the observation period, and potentially consider clinical trials to validate these findings in humans.

## Conclusion

This study evaluated the effects of two vasectomy procedures on male SD rats, focusing on morphology, hormone levels, oxidative stress, sexual behavior, and anxiety. The modified procedure showed significant benefits in reducing tissue damage, apoptosis, and oxidative stress while maintaining endocrine function and improving sexual activity. Future research should investigate long-term effects, the adaptability of this technique across age groups, and ways to minimize physiological and psychological impacts, aiming for safer and more effective male contraceptive options.

## Data Availability

The datasets used or analyzed during the current study are available from the corresponding author upon reasonable request.

## Abbreviations

ASA: Anti-sperm Antibodies
Bax: Bcl-2 Associated X Protein
Bcl-2: B-cell Lymphoma 2
E2: Estradiol
EPM: Elevated Plus Maze
FSH: Follicle Stimulating Hormone
H&E: Hematoxylin and Eosin
HE4: Human Epididymis Protein 4
IL-1: Interleukin-1
IL-2: Interleukin-2
IL-6: Interleukin-6
LH: Luteinizing Hormone
MDA: Malondialdehyde
PGE2: Prostaglandin E2
PRL: Prolactin
SD: Sprague–Dawley
SPF: Specific Pathogen Free
5-HT: Serotonin
SOD: Superoxide Dismutase
T: Testosterone
TEM: Transmission Electron Microscopy
TUNEL: Terminal Deoxynucleotidyl Transferase-mediated dUTP Nick-End Labeling
